# Ginger (*Zingiber officinale*) Attenuates Obesity and Adipose Tissue Remodeling in High-Fat Diet-Fed C57BL/6 Mice

**DOI:** 10.3390/ijerph18020631

**Published:** 2021-01-13

**Authors:** Seok Hee Seo, Feng Fang, Inhae Kang

**Affiliations:** 1 Department of Food Science and Nutrition, Jeju National University, Jeju 63243, Korea; bossni3@jejunu.ac.kr (S.H.S.); fengfang261@gmail.com (F.F.); 2 Interdisciplinary Graduate Program in Advanced Convergence Technology and Science, Jeju National University, Jeju 63243, Korea

**Keywords:** ginger, obesity, adipocyte remodeling

## Abstract

Obesity is characterized by excessive fat accumulation in adipose tissue, which is an active endocrine organ regulating energy metabolism. Ginger (*Zingiber officinale*) is known to have antioxidant, anti-inflammatory, and antiobesity effects, but the role of ginger in modulating adipocyte metabolism is largely unknown. In this study, we hypothesized that ginger supplementation inhibits high-fat (HF)-diet-mediated obesity. C57BL/6 male mice were randomly assigned to three diets for 7 weeks: low fat (LF, 16% kcal from fat), HF (HF, 60% kcal from fat), or HF with 5% ginger powder in diet (HF + G). The HF diet increased body weight (BW) and BW gain, as well as fasting glucose, total cholesterol, and hepatic lipid levels, compared to the LF diet-fed group. Ginger supplementation significantly improved HF-diet-induced BW gain, hyperglycemia, hypercholesterolemia, and hepatic steatosis without altering food intake. Next, we investigated whether ginger modulates adipocyte remodeling. HF-mediated adipocyte hypertrophy with increased lipogenic levels was significantly improved by ginger supplementation. Furthermore, the HF+G group showed high levels of the fatty-acid oxidation gene, carnitine palmitoyltransferase 1 (CPT1), which was accompanied by a reduction in adipocyte inflammatory gene expression. Taken together, our work demonstrated that ginger supplementation attenuated HF-diet-mediated obesity and adipocyte remodeling in C57BL/6 mice.

## 1. Introduction

Obesity, which has reached epidemic proportions world-wide, can cause a number of complications (elevated blood pressure, dyslipidemia, and insulin resistance etc.) [1,2]. Excessive fat accumulation is characterized by obesity and occurs when energy consumption is higher than energy expenditure [3]. Many ongoing studies are actively studying mechanisms to decrease energy overconsumption and increase energy dissipation to prevent energy imbalance. High consumption of nutrient-dense food such as a high-fat (HF) diet or a high-sugar diet induces oxidative stress and mitochondrial damage which results in chronic low-grade inflammation status in the body [4,5]. Unhealthy, obese individuals have not only tried long-term plans for weight loss such as a modification of lifestyle, but also the surgical and/or medicinal interventions [6]. Although there are several available Food and Drug Administration (FDA)-approved pharmaceuticals for combating obesity, its prevalence still remains high.

Previously, adipose tissue was considered to be a simple energy reservoir; however, it is currently regarded as a tissue regulating whole-body metabolism [7]. In response to energy overload, adipocytes dynamically undergo remodeling, thereby altering the adipocyte number/size and stromal vascular-cell recruitment in adipose tissue, including immune cells. These events could cause the dysregulation of adipose tissue, such as adipocyte death (efferocytosis), adipogenesis, or angiogenesis. This series of events is called “adipose tissue remodeling” [8,9,10]. Although substantial efforts are being made to develop lead candidates to prevent aberrant adipocyte remodeling, many side-effects have been reported. Thus, highly effective and safe drugs or components with a low number of adverse effects, such as bioactive nutraceuticals, for controlling abnormal adipocyte turnover could be a strategy for preventing obesity.

Ginger (*Zingiber officinale*) is a herb belonging to the ginger family (Zingiberaceae) of subtropical/tropical origin, widely used as a spice and flavoring material, especially in Asia. Ginger contains various physiologically active nutrients, including phenolic compounds such as gingerols and shogaols and active components such as flavonoids and terpenoids [11]. Recent evidence shows that these components in ginger have health-promoting effects [12,13,14,15]. 6-gingerol, responsible for the unique taste of ginger has been reported to exhibit anti-inflammatory, antiseptic, and antioxidant activities [16], and gingerol and shogaol have been investigated to enhance immunity [17,18]. In recent years, various physiological effects, including the antiobesity effects of ginger and several bioactive components in ginger (gingerol and gingerone A etc.) have been revealed in ginger supplementation in vivo [19,20,21,22]. Furthermore, systematic reviews of recent clinical trials with ginger supplementation reported that ginger supplementation resulted in a remarkable reduction in low-density lipoprotein cholesterol (LDL-C), total cholesterol (TC), and triglyceride (TG) levels, as well as an increase in high-density lipoprotein cholesterol (HDL-C) concentration [23,24]. However, the effect of ginger on adipocyte metabolism in vivo is still unknown.

The objective of this study is to explore the role of ginger on HF-diet-mediated obesity and adipose remodeling. We postulated that ginger flour supplementation could reduce high-fat (HF)-diet-mediated body weight (BW) gain, dyslipidemia, fatty liver, and adipocyte remodeling. To address this hypothesis, HF (60% kcal from fat) diet-fed C57BL/6 mice were used as an animal model and 3T3-L1 adipocytes were used as an in vitro model to investigate the potential role of ginger supplementation in obesity and adipocyte remodeling, as well as its possible mechanisms.

## 2. Materials and Methods

### 2.1. Experimental Materials

The ginger powder was purchased from the PRDP (Seoul, Korea). Most cell cultures were purchased from SPL (Seoul, Korea). Dulbecco’s Modified Eagle Medium (DMEM), fetal bovine serum (FBS), and penicillin/streptomycin were purchased from Gibco (Grand Island, NY, USA). All other chemicals and reagents were purchased from Sigma Chemical Co. (St. Louis, MO, USA) unless otherwise stated.

The ginger powder was extracted using the pressurized hot water extraction method (modified from [25]) as previously described. The final stock concentration of ginger was prepared at 100 mg/mL with several aliquots for in vitro experiments.

### 2.2. Animals and Diets

All protocols and procedures were approved by the Institutional Animal Care and Use Committee at Jeju National University (Approval ID # 2018-0012). Male 5-week-old C57BL/6J mice were purchased from the ORIENT BIO Animal Center (Seongnam-si, Korea) and housed in a dark/light cycle at Jeju National University. Mice at 6 weeks of age were randomly assigned to one of three experimental groups fed with different diets *ad libitum* for 7 weeks: low-fat diet (16% kcal from fat, LF group, *n* = 4), high-fat diet (60% kcal from fat, HF group, *n* = 5), or HF diet mixed with 5% of ginger (5 g ginger powder/kg diet, HF + G group, *n* = 5). For the HF + G diet, cellulose was substituted for ginger powder. The AIN93G diet was used for LF diet control, and HF diet formulation was adapted from a typical 60% kcal% fat diet [26] (Table 1). The daily *ad libitum* food intake per mouse was measured for 3 days in the last week of feeding. Body weight (BW) was monitored every week throughout the study. The BW gain of each experimental group was calculated by subtracting the body weight before the start of the experiment from the final body weight (%) with area under the curve (AUC) [27]. The feeding efficiency ratio (FER, %) was obtained by dividing the weight gain by the dietary intake during the same period.

### 2.3. Measurement of Blood Biochemical Parameters and Hepatic TG Content

After completion of the experiment, the animals were fasted for 12 h and were sacrificed by carbon dioxide narcosis. Blood was collected from a cardiac puncture, and serum samples were aliquoted. Serum total cholesterol (TC, mg/dL) was analyzed using an enzyme assay kit (Asan Pharmaceutical Co., Seoul, Korea) with absorbance measured at 500 nm. Fasting glucose concentration (mg/dL) was measured using a blood glucose meter (Midium Blood Glucose Analyzer, Kia Ace Co., Ltd., Gyeonggi, Korea).

To measure triglyceride content (TG, mg/dL) in liver, lipid extraction from ~0.2 g of liver was performed using a chloroform/methanol (2:1) solution and processed as described previously [28]. TG was analyzed using an enzyme assay kit (Asan Pharmaceutical Co., Seoul, Korea) and normalized by protein (mg TG/g protein).

### 2.4. Hematoxylin and Eosin (H&E) Staining and Adipocyte Size Measurement

Upon sacrificing of mice, liver and adipose tissues were dissected and immediately fixed in 10% buffered formalin for histology assessment. Fixed samples were dehydrated in graded alcohol, and embedded in paraffin. Paraffin-embedded blocks were sectioned into thickness of 5–7 μm. Sections were deparaffinized in xylene, dehydrated with graded alcohol, and processed for hematoxylin and eosin (H&E) staining as described previously [28]. Bright-field images were obtained by a Leica microscope (Leica DM 2500, Leica, IL, USA) under 20× magnification. H&E sections of epididymal adipose tissue were used for size determination/quantification. Adipocyte size was quantified using Image J and Adiposoft from the National Institutes of Health (NIH).

### 2.5. Total Polyphenol and Flavonoid Contents of Ginger Extract

The total polyphenol and flavonoid contents of the ginger extract were analyzed using the modified Folin–Ciocalteu and aluminum chloride method as previously described [29]. Total polyphenol and flavonoid results were expressed as gallic acid concentration equivalents and catechin equivalents, respectively.

### 2.6. Cell Culture and Cell Viability Assay

The 3T3-L1 cells (ATCC^®^ CL-173™, Manassas, VA, USA) were cultured in basal medium (DMEM with penicillin/streptomycin and 2 mM l-glutamine (Sigma Chemical Co., St. Louis, MO, USA) supplemented with 10% newborn calf serum (Linus, Madrid, Spain) as described previously [29]. The 3T3-L1 cells were seeded into six-well plates and cultured until they reached confluence. After 2 days, when the cells reached confluence (referred to as day 0), 3T3-L1 cells were induced to differentiation in a basal medium containing 10% FBS (Gibco, Grand Island, NY, USA), 1 μM dexamethasone (Sigma), 0.5 mM 3-isobutyl-1-methylxanthine (Sigma), and 2 nM insulin (Sigma) for 48 h. Then, cells were cultured in basal medium containing 10% FBS with 2 nM insulin for 2 days and without insulin for an additional 7–10 days. To measure the impact of ginger extract on lipid accumulation in adipocytes, cells were treated with ginger extract during differentiation until day 7, then stained with Oil Red O (ORO) as described previously [29].

2,3-Bis-(2-methoxy-4-nitro-5-sulfophenyl)-2H-tetrazolium-5-carboxanilide salt (XTT) assay was carried out to determine the cell viability of 3T3-L1 cells after treatment of ginger extract. Briefly, 3T3-L1 cells were cultured in 96-well plates and incubated with or without ginger extract (15–120 μg/mL). After 24 h, the cell medium was replaced with a fresh medium containing with XTT reagent for 3 h. Cell viability was measured at 450 nm using a microplate reader according to the manufacturer’s protocol (Cell Signaling Technology, Danvers, MA, USA).

### 2.7. Analysis of Messenger RNA (mRNA) by Real-Time Polymerase Chain Reaction (RT-PCR)

After the end of the experiment, 0.1 g of hepatic tissue or 0.2 g of epididymal fat tissue, which was stored in a freezer, was subjected to RNA extraction using Trizol reagent (Invitrogen Co., Carlsbad, CA, USA). First, 1–2 μg of RNA was converted into complementary DNA (cDNA) using high-capacity cDNA reverse-transcription kits (Applied Biosystem, USA). Relative gene expression, which was normalized to hypoxanthine–guanine phosphoribosyl transferase (HPRT) and/or ribosomal protein lateral stalk subunit P0 (RPLP0, 36B4) (Cosmo Genetech, Table 2), was determined by real-time PCR (CFX96™ Real-Time PCR Detection System, Bio-Rad, Hercules, CA, USA).

### 2.8. Protein Isolation and Western Blotting

Harvested tissue samples were homogenized with a homogenizer in radioimmunoprecipitation assay (RIPA) lysis buffer (Thermo Fisher Scientific, Waltham MA, USA) with a protease and phosphatase inhibitor cocktail (Sigma) and centrifuged to collect the supernatant. Twelve micrograms of protein were fractionated by 10% SDS-PAGE and transferred to a polyvinylidene difluoride (PVDF) membrane (Thermo Fisher Scientific, MA, USA). Primary antibodies against fatty acid synthase (Fas), peroxisome proliferator-activated receptor γ (PPARγ), and β-actin were obtained from Cell Signaling Technology (Danvers, MA, USA). Antibodies against adipocyte protein 2 (aP2, FABP4) were purchased from Santa Cruz biotechnology (Santa Cruz, CA, USA). To identify the blot, ChemiDoc (Bio-Rad, CA, USA) with enhanced chemiluminescence (ECL) reagent (PerkinElmer, Waltham, MA, USA) was used, and the expression level was calculated using Image J (NIH, Bethesda, MD, USA).

### 2.9. Statistics

Data are presented as the mean ± standard error of the mean (SEM). Samples were statistically evaluated using one-way ANOVA (analysis of variance) with Bonferroni’s multiple comparison test or Student’s *t*-test. GraphPad Prism 7.0 (La Jolla, CA, USA) was used for statistical analysis.

## 3. Results

### 3.1. Ginger Reduced Lipid Accumulation in 3T3-L1 Adipocytes

**Figure 1:**
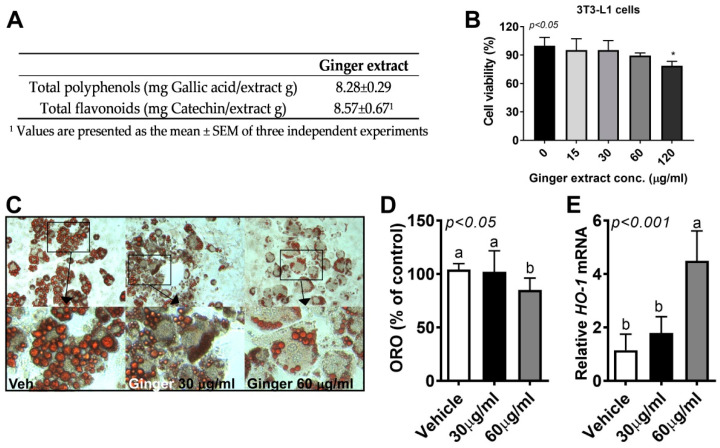
Ginger reduced lipid accumulation in 3T3-L1 adipocytes. (**A**) Total polyphenol and flavonoid contents of ginger extract; the culture of 3T3-L1 cells was incubated with ginger extract at different doses (15–120 μg/mL). 2,3-Bis-(2-methoxy-4-nitro- 5-sulfophenyl)-2H-tetrazolium-5-carboxanilide salt (XTT) reagent was added for 3 h into 96-well plates to measure cell viability. (**B**) Cell viability by XTT; the 3T3-L1 cells were differentiated in the presence or absence of ginger extract (0–60 μg/mL) for 7 days. Dimethyl sulfoxide (DMSO) was used as a vehicle control. (**C**) Triglyceride (TG) accumulation in 3T3-L1 adipocytes visualized using Oil Red O (ORO) staining. Representative images from three separate experiments are shown (20× magnification, upper). Images were cropped to the relevant part of the field without altering the resolution (close-cropped images, lower). (**D**) ORO dye was extracted using isopropanol and quantified as the optical density at 500 nm (OD_500_). (**E**) Heme oxygenase 1 (*HO-1*) gene expression determined using real-time PCR. Data are represented as the mean ± standard error of the mean (SEM) of three independent experiments. * *p* < 0.05 (compared with vehicle). Bars with different letters are significantly different according to one-way ANOVA with Bonferroni’s comparison test.

We firstly determined the polyphenol and flavonoid contents in ginger. The ginger extract was found to contain 8.28 mg gallic acid/g extract of total polyphenol content, while the total flavonoid content of ginger extract was equivalent to 8.57 mg catechin/g extract (Figure 1A).

Next, to determine whether ginger is a negative regulator of adipocyte hyperplasia, we first investigated the cytotoxic effects of ginger extract (15–120 μg/mL) in 3T3-L1 adipocytes. Ginger extract was treated 24 h before the cell viability assay and the 2,3-Bis-(2-methoxy-4-nitro-5-sulfophenyl)-2H-tetrazolium-5-carboxanilide salt (XTT) assay was performed. There was a significant reduction in cell viability with 120 μg/mL of ginger extract, but not with 15–60 μg/mL (Figure 1B). Ginger extract (30–60 μg/mL) was added to 3T3-L1 cells during adipocyte differentiation and maintained for 7 days. The presence of 60 μg/mL ginger extract caused a significant reduction in TG accumulation as measured by Oil Red O (ORO) staining (Figure 1C,D). Furthermore, the gene expression of heme oxygenase 1 (*HO-1*), a potent antioxidant that plays a crucial role in decreasing reactive oxygen species (ROS), was significantly upregulated by 60 μg/mL ginger extract (Figure 1E). This implies that the reduction in lipid accumulation in adipocytes might be related to the antioxidant effects of ginger extract.

### 3.2. Ginger Supplementation Ameliorated HF-Diet-Induced Metabolic Parameters without a Change in Food Intake

**Figure 2:**
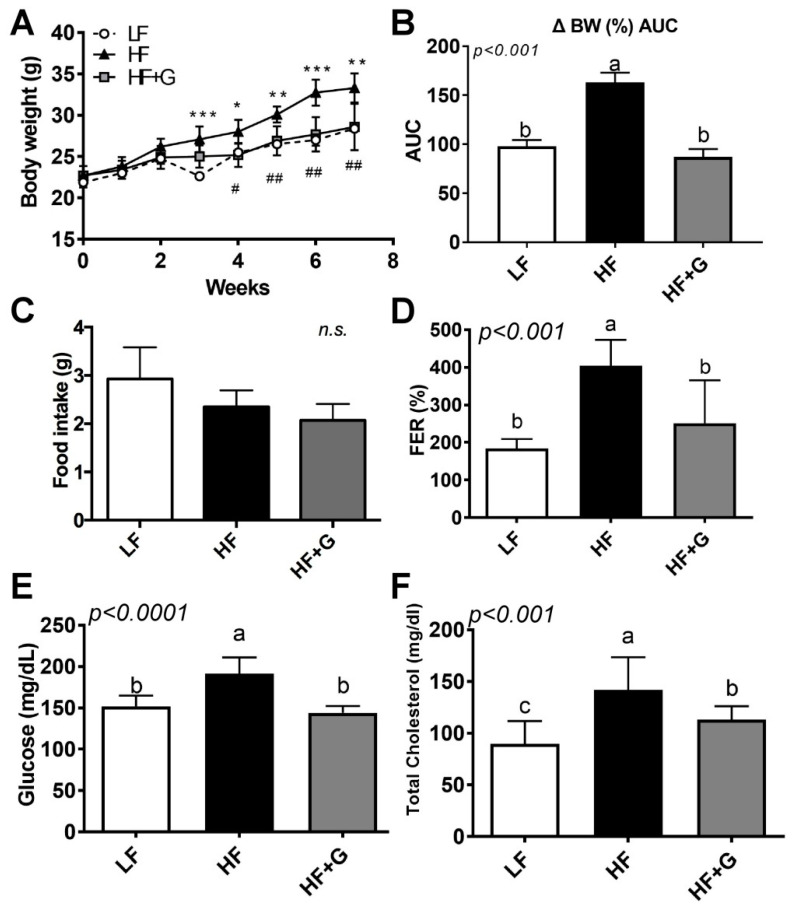
Ginger supplementation ameliorated HF-diet-induced metabolic parameters without a change in food intake. Male 6-week-old C57BL/6 mice were fed an LF (white circle, white bar), HF (black triangle, black bar), or HF + G (gray square, gray bar) diet for 7 weeks (*n* = 4–5 per group). (**A**) body weight (g); (**B**) body weight (%) AUC (BW gain throughout the 7 weeks experiment expressed as % change from week 0 with AUC); (**C**) food intake (g/day); (**D**) feeding efficiency ratio (FER, %), calculated as body weight gain (g/day) divided by food intake (g/day); (**E**) fasting glucose levels (mg/dL); and (**F**) fasting serum total cholesterol levels (mg/dL). Data are expressed as the mean ± SEM (*n* = 4–5). n.s. represents no significance. Bars with different letters are significantly different according to one-way ANOVA with Bonferroni’s comparison test; * *p* < 0.05, ** *p* < 0.01, *** *p* < 0.001 (LF vs. HF); ^#^ *p* < 0.05, ^##^ *p* < 0.01 (HF vs. HF + G).

Next, we hypothesized that ginger supplementation would reverse HF-diet-mediated obesity. To address this hypothesis, C57BL/6 mice were fed a HF diet supplemented with ginger (5% ginger in HF diet) and compared to those fed a high-fat diet (HF) alone or a low-fat diet (LF). We then investigated their BW, BW gain (BWG), food intake, and feeding efficiency ratio (FER). The mean BW before feeding was 22.46 ± 0.23 g, as shown in Figure 2A. After 7 weeks on the various diets, the final body weight was 28.38 ± 0.24 g in the LF group, 33.3 ± 0.78 g in the HF group, and 28.6 ± 1.26 g in the HF + G group. BW gain was also significantly decreased in the ginger intake group compared to the HF group (Figure 2B, *p* < 0.01). There was no significant difference in dietary intake among the groups (Figure 2C). The feeding efficiency ratio was calculated as a function of BW change and dietary intake. The energy utilization rate in the HF + G group was significantly lower than that in the HF group (Figure 2D).

To investigate the effect of ginger on glucose control, mice were fasted for 12 h, and blood glucose levels were checked in the veins of the tail. The HF diet significantly increased fasting plasma glucose level (LF, 152 ± 6.42 mg/dL; HF: 191.4 ± 8.78 mg/dL), which was significantly decreased upon the intake of ginger (HF + G: 144 ± 4.73 mg/dL, *p* < 0.0001; Figure 2E). Furthermore, Figure 2F showed the level of total cholesterol (TC) in blood, which was significantly decreased in the HF + G group compared to the HF group (89.52 ± 7.86 mg/dL in the LF group, 141.8 ± 11.97 mg/dL in the HF group, and 113.2 ± 4.29 mg/dL in the HF + G group, *p* < 0.001). These data suggest that ginger supplementation resulted in weight loss with improved blood glucose and lipid profiles.

### 3.3. Ginger Supplementation Reduced HF-Diet-Induced Hepatic Lipid Accumulation

**Figure 3:**
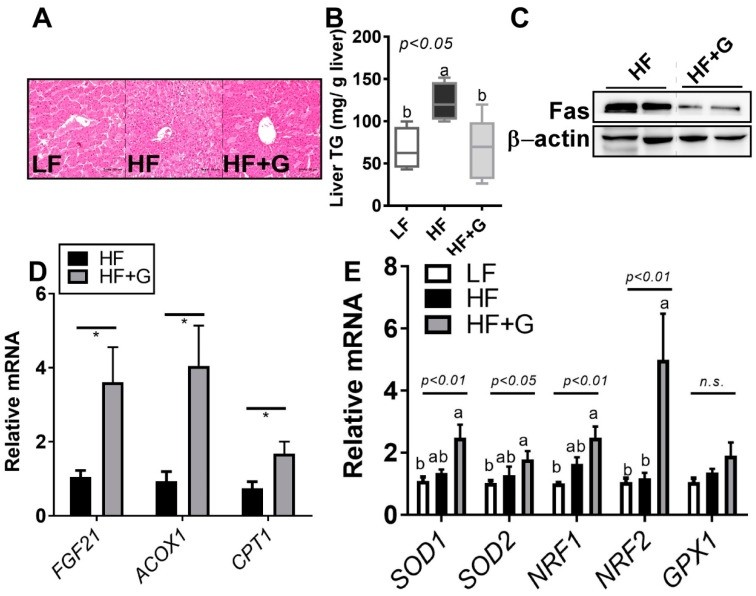
Ginger supplementation reduced HF-diet-induced hepatic lipid accumulation. Male 6-week-old C57BL/6 mice were fed an LF (white), HF (black), or HF + G (gray) diet for 7 weeks (*n* = 4–5 per group). (**A**) Hematoxylin and eosin (H&E) staining of liver tissue (20× magnification); (**B**) hepatic TG content (mg/g liver); (**C**) hepatic fatty-acid synthase (Fas) protein expression; (**D**) hepatic messenger RNA (mRNA) expression of fibroblast growth factor 21 (FGF21), acyl-CoA oxidase 1 (ACOX1), and carnitine palmitoyltransferase 1 (CPT1) determined using real-time PCR; and (**E**) superoxide dismutase 1 (SOD1), SOD2, nuclear factor erythroid 2-related factor 1 (NRF1), NRF2, and glutathione peroxidase 1 (GPX1) gene expressions quantified by real-time PCR. Data are expressed as the mean ± SEM (*n* = 4–5). n.s. represents no significance. Bars with different letters are significantly different according to one-way ANOVA with Bonferroni’s comparison test; * *p* < 0.05 (HF vs. HF + G) with Student’s *t*-test.

To determine the impact of ginger on hepatic lipid metabolism, hepatic histopathology was investigated using H&E staining. H&E staining showed a reduction in hepatic lipid accumulation in the HF vs. HF + G group (Figure 3A). Hepatic histological changes were confirmed by the hepatic TG content. As expected, the HF diet induced an approximately 1.8-fold increase in hepatic lipid accumulation, which was significantly reduced (~50%) by ginger supplementation (Figure 3B). Consistent with these results, hepatic fatty-acid synthase (Fas), which is major protein for de novo lipogenesis of long-chain fatty acids, was reduced by ginger supplementation (Figure 3C). These events were accompanied by the upregulation of fatty-acid (FA) oxidation genes such as fibroblast growth factor 21 (FGF21), acyl-CoA oxidase 1 (ACOX1), and carnitine palmitoyltransferase 1 (CPT1) upon ginger intake (Figure 3D). Interestingly, ginger supplementation upregulated antioxidant enzymes involved in reactive oxygen species elimination, including superoxide dismutase (SOD) 1/2, nuclear factor erythroid 2-related factor (NRF) 1/2, and glutathione peroxidase (GPX), which are involved in the primary defense against oxidative damage (Figure 3E). This suggests that the ginger-induced decrease in hepatic lipid accumulation is, at least in part, related to an augmentation in hepatic FA oxidation and hepatic antioxidant defense mechanisms.

### 3.4. Ginger Supplementation Attenuated HF-Induced Adipocyte Hypertrophy

**Figure 4:**
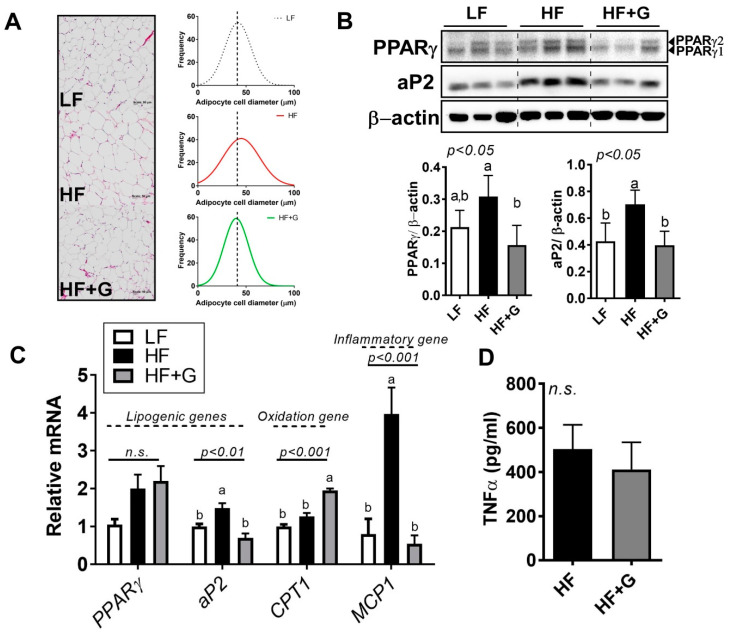
Ginger supplementation attenuated HF-induced adipocyte hypertrophy. Male 6-week-old C57BL/6 mice were fed an LF (white), HF (black), or HF + G (gray) diet for 7 weeks (*n* = 4–5 per group). (**A**) H&E staining of adipose tissue (20× magnification) and adipocyte size distribution (Gaussian curve fitting). (**B**) protein expression of peroxisome proliferator-activated receptor γ (PPARγ) and adipocyte protein 2 (aP2) in epididymal adipose tissue, quantified by Western blot. β-Actin was used as a loading control. (**C**) mRNA expression of *PPARγ*, *aP2*, *CPT1*, and *MCP1* in epididymal adipose tissue determined using real-time PCR. (**D**) TNFα cytokine production by ELISA. Data are expressed as the mean ± SEM (*n* = 4–5). n.s. represents no significance. Bars with different letters are significantly different according to one-way ANOVA with Bonferroni’s comparison test.

Next, we raised the question of whether ginger alters adipocyte energy metabolism. Morphological changes in visceral fat were examined using H&E staining of epididymal fat. Figure 4A (left) shows that adipocytes were smaller in size in the HF + G group compared to the HF group. Next, epididymal adipocyte size was examined by analyzing digital images of H&E sections using software. The HF-diet-mediated expansion in adipocyte size was shifted toward a smaller level upon ginger supplementation, similar to the level seen for the LF group (Figure 4A, right). The reduction in adipocyte hypertrophy was accompanied by attenuation of PPARγ, which is a major transcription factor that regulates differentiation at the early stages of adipocyte differentiation, and aP2, which is the PPARγ target protein (Figure 4B). Although ginger supplementation did not reduce *PPARγ* mRNA expression compared to the HF group, the expression of the *aP2* (*FABP4*) gene was significantly reduced by ~50%. In contrast, *CPT1* gene expression was considerably enhanced by ginger compared to the HF group (Figure 4C).

Adipocyte expansion is followed by low-grade inflammation during adipose tissue remodeling [30]. We found that monocyte chemotactic protein-1 (MCP1), a proinflammatory chemokine that serves as a potent adipokine, was reduced in the ginger-supplemented group (Figure 4C, right). Furthermore, there was a marginal but nonsignificant decrease in systemic levels of tumor necrosis factor α (TNFα) production (Figure 4D). These results indicated that ginger ameliorated HF-mediated hypertrophy and adipose inflammation via the upregulation of fatty-acid oxidation.

## 4. Discussion

The present study demonstrated that ginger, which has a high number of bioactive components (Figure 1A), suppressed HF-induced metabolic parameters such as BWG, fasting glucose, total cholesterol levels (Figure 2), and hepatic steatosis (Figure 3). Furthermore, ginger altered HFD-mediated adipose tissue remodeling by reducing adipose tissue size and adipose inflammation (Figure 4). Ginger also reduced adipogenic conversion during adipogenesis (Figure 1C,D). These antiobesity effects of ginger might partly involve the induction of antioxidant effects (Figure 1E and Figure 3E) and fatty-acid oxidation (Figure 3D and Figure 4C).

It has been well demonstrated that ginger possesses antiobesity properties, leading to a reduction in BW and fat mass in various animal models [31,32,33,34]. Moreover, ginger was shown to be effective in reducing cardiovascular disease risk by ameliorating dyslipidemia in humans [23,35]. In our study, we also found that ginger flour (5%) added to an HF diet (cellulose was substituted with ginger powder) reduced HF-diet-mediated BWG, hyperglycemia, and hypercholesterolemia (Figure 2), in alignment with the recent study [31] showing that ginger water treatment for 4 weeks significantly reduced BW, TG, and TC contents, as well as altered the transcriptional level of energy-metabolizing proteins, in hepatic and adipose tissue. In both in vivo and clinical studies, the consumption of steamed ginger ethanolic extract, with a high amount of 6-shogaol, by healthy obese people (12 week, randomized, double-blind, placebo-controlled clinical trial) led to a significant reduction in BW and body fat [35]. The authors also mentioned that ginger supplementation might result in an increase in the efficacy of physical activity. In our study, hepatic *ACOX1*, *CPT1*, and *PGC1α* levels (Figure 2), as well as adipose tissue *CPT1* levels (Figure 3), which are associated with energy expenditure, were upregulated by ginger supplementation. This implies that the ginger-induced reduction in BWG and fat size distribution may involve the regulation of energy expenditure. Further studies should investigate the association between BW and energy expenditure. Although 7 weeks of ginger supplementation in HF-fed mice was enough to reduce obesity and adipose remodeling, 7 weeks of HF diet is considered too short to identify the impact of food matrices or components in obesity. We are designing another experiment, using the long-term HF diet-fed mice (more than 12-16 weeks) to investigate the impact of ginger consumption on obesity-mediated metabolic complications (such as hepatic steatosis, and diabetes etc.).

Hepatic steatosis can be caused by several factors such as diet control (HF and/or high-sucrose diet), insulin resistance, and genetic mutation [36,37]. Ginger supplementation significantly attenuated HF-diet-mediated hepatic lipid accumulation by ~50%, which may have involved the upregulation of fatty-acid oxidation, as well as antioxidant mechanisms, in the liver (Figure 2). Hepatic lipid overload induces the overproduction of oxidants by affecting several reactive oxygen species (ROS)-generating mechanisms [38]. A high antioxidant status is related to protective capacities against the high oxidative stress featured in nonalcoholic fatty liver disease (NAFLD). Sixteen weeks of ginger fed orally in an HFD-supplemented animal model led to reduced liver steatosis and low-grade inflammation, along with modulation of the gut microbiota composition [33]. Furthermore, 200 mg/kg steamed ginger extract, with a high antioxidant capacity, was also shown to instigate a decrease in hepatic lipids and hepatic lipogenesis/lipolysis genes compared to the HF group [34]. Although we did not measure enzymatic antioxidant activity in the liver (or systemic levels), it is plausible that the prevention of HF-diet-induced oxidative stress in the liver may lead to an attenuation of hepatic steatosis. Future work will involve the organelle-targeted measurement of antioxidant capacities, such as in the mitochondria or endoplasmic reticulum (ER), to answer these unsolved research questions.

The function of adipose tissue as a systemic energy regulator in the control of whole-body homeostasis has been extensively investigated by many researchers [8,9]. In excess nutrient conditions, adipocyte hypertrophy leads to an increase in immune cells and inflammatory cytokines [39], which induce a chronic inflammation state during the secretion of insulin, thereby leading to metabolic defects such as insulin resistance, type 2 diabetes, and cardiovascular diseases [8,30,39]. In our study, we showed that adipocyte number (hyperplasia, Figure 1C,D) and adipocyte size (hypertrophy, Figure 4A) were reduced by ginger supplementation, along with a reduction in adipocyte inflammation (Figure 4C). This is consistent with recent evidence showing that ginger extracted using high hydrostatic pressure triggered a reduction in adipocyte size, along with the attenuation of adipose tissue inflammation [34]. As mentioned previously, we hypothesized that the ginger-induced reduction in body fat may be due to mechanisms involving energy expenditure. Several recent studies reported that ginger and its major bioactive component, 6-shogaol, were effective in brown adipose tissue differentiation and white adipocyte browning [16], induced by heat [32]. Although we did not perform adipocyte browning experiments in this study, we demonstrated that a marker specific for fatty-acid oxidation (CPT1) was significantly enhanced by ginger supplementation compared to the HF and LF groups (Figure 4C). Moreover, the improvement in insulin sensitivity evidenced by the reduction in fasting glucose (utilization of glucose, Figure 2B) may have also been affected by the increase in FA oxidation, leading to changes in adipocyte size distribution (Figure 4A). Changes in visceral adiposity followed by adipose tissue inflammation were also found in the ginger-supplemented group, as evidenced by the significant reduction in *MCP1* gene expression in adipose tissue (Figure 4C). However, according to our findings, it is plausible that the induction of energy expenditure, especially through the mitochondria (upregulation of FA oxidation (Figure 3 and Figure 4) and brown adipose tissue activation [32,40]), may contribute to the adipocyte remodeling process triggered by ginger and/or its major components such as shogaol and gingerol. It is still unclear, however, whether ginger directly targets adipose tissue metabolism or systemically targets whole-body metabolism. Furthermore, adipose tissue browning by ginger was not tested in our study. However, future work will involve testing uncoupling protein 1 (UCP1)-mediated heat generation by ginger using a thermogenic animal model exposed to the cold and/or using a β-adrenergic receptor agonist. Moreover, the tissue levels of ginger and/or its bioactive components require further investigation.

Altogether, our results show that 5% ginger flour intake reduced obesity and adipose tissue remodeling via (i) weight loss, (ii) an improvement in blood glucose/lipid level, (iii) hepatic steatosis via an upregulation of fatty-acid oxidation, and (iv) a reduction in visceral adiposity and adipose inflammation, potentially involving ginger’s antioxidant properties. One of the limitations of our study is that we found a slight reduction of body weight in the LF diet (at the third week, Figure 2A). This may have been due to unexpected fasting, fighting against each other, or behavior issues. Although it is still vague, weight loss occurred during the experimental period and final BW and BWG were significantly different between HF and HF+G groups (Figure 2A,B). Moreover, the minimum number of mice per group that we calculated was five animals/group [41]: HF and HF+G group meet the criteria, but not LF (n = 4). Repeating an animal experiment is necessary to improve accuracy and precision. Some research questions remain unsolved following our study: (i) What components are responsible for the antiobesity effects of ginger? (ii) Are there any metabolites following the consumption of ginger and what is their absorption rate, especially in adipose tissue? (iii) What specific mechanisms are involved in this overall reduction in obesity and adipose tissue remodeling? (iv) Although 5% ginger supplementation for 7 weeks was enough to reduce BW and adipose expansion, is it physiologically achievable in humans (in terms of duration, quantity, and efficacy)? To answer these questions, future work will involve experiments investigating tissue-specific ginger absorption and mechanistic studies using animals.

In spite of the missing links mentioned above, our work provides additional insights into ginger’s properties with respect to diminishing nutrient overload-mediated metabolic complications, as well as adipose tissue remodeling, which involves, at least in part, protective mechanisms against oxidative stress and fatty-acid oxidation.

## 5. Conclusions

The current study was designed to investigate whether ginger could ameliorate HF-diet-induced obesity and adipose tissue remodeling in C57BL/6 mice. In our study, the HF diet significantly induced BWG, hyperglycemia, hypercholesterolemia, hepatic steatosis, and white adipose tissue expansion. We found that 5% ginger flour intake for 7 weeks dramatically improved the abovementioned metabolic complications via the induction of fatty-acid oxidation and the prevention of oxidative stress induced by HFD. Taken together, our work highlights ginger as a potentially promising dietary strategy to prevent HF-mediated obesity and adipose tissue remodeling.

## Figures and Tables

**Table 1:** Dietary composition of low-fat (LF), high-fat (HF), and HF plus 5% ginger powder (HF + G) diets ^1^.

Ingredients	LF	HF	HF + G
	g/kg	g/kg	g/kg
Casein	200	200	200
l-Cysteine	3	3	3
Sucrose	100	68.8	68.8
Corn starch	397	0	0
Maltodextrin 10	132	125	125
Lard	0	245	245
Soybean oil	70	25	25
Cellulose	50	50	0
Mineral mix	35	35	35
Calcium phosphate	3.4	3.4	3.4
Vitamin mix	10	10	10
Choline bitartrate	2.5	2	2
Ginger powder	0	0	50
Total	1002.9	767.2	767.2
	kcal (%)	kcal (%)	kcal (%)
Carbohydrate	63	19	19
Protein	20	20	20
Fat	16	60	60

^1^ The AIN-93G diet was modified.

**Table 2:** Primer sequences for real-time PCR.

Gene	Forward	Reverse
*m36B4*	GGATCTGCTGCATCTGCTTG	GGCGACCTGGAAGTCCAACT
*mACOX1*	CTTGGATGGTAGTCCGGAGA	TGGCTTCGAGTGAGGAAGTT
*maP2*	AGCATCATAACCCTAGATGGCG	CATAACACATTCCACCACCAGC
*mCPT1*	CCAGGCTACAGTGGGACATT	AAGGAATGCAGGTCCACATC
*mFGF21*	GCTCTCTATGGATCGCCTCAC	GGCTTCAGACTGGTACACATT
*mGPX1*	AGTCCACCGTGTATGCCTTCT	GAGACGCGACATTCTCAATGA
*mHO-1*	CAGGTGATGCTGACAGAGGA	TCTCTGCAGGGGCAGTATCT
*mHPRT*	TTGCTCGAGATGTCATGAAGGA	AGCAGGTCAGCAAAGAACTTATAGC
*mMCP1*	AGGTCCCTGTCATGCTTCTG	GCTGCTGGTGATCCTCTTGT
*mNRF1*	CCCTACTCACCCAGTCAGTATG	CATCGTGCGAGGAATGAGGA
*mNRF2*	TCAGCGACGGAAAGAGTATGA	CCACTGGTTTCTGACTGGATGT
*mPGC1* *α*	CCCTGCCATTGTTAAGACC	TGCTGCTGTTCCTGTTTTC
*mPPAR* *γ*	GGCGATCTTGACAGGAAAGAC	CCCTTGAAAAATTCGGATGG
*mSOD1*	AACCAGTTGTGTTGTCAGGAC	CCACCATGTTTCTTAGAGTGAGG
*mSOD2*	CAGACCTGCCTTACGACTATGG	CTCGGTGGCGTTGAGATTGTT
